# Key circRNAs from goat: discovery, integrated regulatory network and their putative roles in the differentiation of intramuscular adipocytes

**DOI:** 10.1186/s12864-023-09141-7

**Published:** 2023-01-28

**Authors:** Du Yu, Li Xin, Xu Qing, Zhang Hao, Wang Yong, Zhu Jiangjiang, Lin Yaqiu

**Affiliations:** 1 Key Laboratory of Qinghai-Tibetan Plateau Animal Genetic Resource Reservation and Utilization, Ministry of Education, Southwest Minzu University, Chengdu, China; 2 Key Laboratory of Qinghai-Tibetan Plateau Animal Genetic Resource Reservation and Exploitation of Sichuan Province, Southwest Minzu University, Chengdu, China; 3 College of Animal and Veterinary Sciences, Southwest Minzu University, Chengdu, China

**Keywords:** NcRNAs, Adipocyte differentiation, Interaction network, Goat

## Abstract

**Background:**

The procession of preadipocytes differentiation into mature adipocytes involves multiple cellular and signal transduction pathways. Recently. a seirces of noncoding RNAs (ncRNAs), including circular RNAs (circRNAs) were proved to play important roles in regulating differentiation of adipocytes.

**Result:**

In this study, we aimed to identificate the potential circRNAs in the early and late stages of goat intramuscular adipocytes differentiation. Using bioinformatics methods to predict their biological functions and map the circRNA-miRNA interaction network. Over 104 million clean reads in goat intramuscular preadipocytes and adipocytes were mapped, of which16 circRNAs were differentially expressed (DE-circRNAs). Furthermore, we used real-time fluorescent quantitative PCR (qRT-PCR) technology to randomly detect the expression levels of 8 circRNAs among the DE-circRNAs, and our result verifies the accuracy of the RNA-seq data. From the Kyoto Encyclopedia of Genes and Genomes (KEGG) enrichment analysis of the DE-circRNAs, two circRNAs, circ_0005870 and circ_0000946, were found in Focal adhesion and PI3K-Akt signaling pathway. Then we draw the circRNA-miRNA interaction network and obtained the miRNAs that possibly interact with circ_0005870 and circ_0000946. Using TargetScan, miRTarBase and miR-TCDS online databases, we further obtained the mRNAs that may interact with the miRNAs, and generated the final circRNA-miRNA-mRNA interaction network. Combined with the following GO (Gene Ontology) and KEGG enrichment analysis, we obtained 5 key mRNAs related to adipocyte differentiation in our interaction network, which are FOXO3(forkhead box O3), PPP2CA (protein phosphatase 2 catalytic subunit alpha), EEIF4E (eukaryotic translation initiation factor 4), CDK6 (cyclin dependent kinase 6) and ACVR1 (activin A receptor type 1).

**Conclusions:**

By using Illumina HiSeq and online databases, we generated the final circRNA-miRNA-mRNA interaction network that have valuable functions in adipocyte differentiation. Our work serves as a valuable genomic resource for in-depth exploration of the molecular mechanism of ncRNAs interaction network regulating adipocyte differentiation.

**Supplementary Information:**

The online version contains supplementary material available at 10.1186/s12864-023-09141-7.

## Introduction

Intramuscular fat (i.m.) content is a determinant of edible flavor of the meat of farm animals. Unlike subcutaneous and visceral fat content, in the livestock industry, higher levels of intramuscular fat content have been shown to help improve flavor and palatability of meat [1, 2]. Adipogenesis consists of two phases, namely commitment and terminal differentiation. Preadiposcyte (brown or white or brite) presenting throughout adult life, it can differentiate and proliferate from distinct progenitor cells [3]. Examine the signaling cascades and regulation behind adipogenesis and adiposcyte differentiation, may provide insight into the plasticity of adipose tissue and the development of new techniques in animal husbandry. Numerous studies have demonstrated that adipocyte formation and deposition is a complex and precisely orchestrated process that mediated by a series of adipocyte regulatory factor networks. In it, the roles of ncRNAs in regulatory adipocyte differentiation, proliferation and fat deposition cannot be ignored [4–7]. However, the endogenous regulatory pathways and functions for most ncRNAs are still unclear. Thus, summarizing and mining new interactive networks is of substantial significance to explore the regulatory mechanisms of intramuscular adipocyte differentiation and deposition.

Benefiting from the development of high-throughput sequencing and computer analysis techniques, the classifications of ncRNAs are becoming more specific. Nowadays, it has been realized to identified differentially enriched ncRNAs in tissues of different growth conditions or different developmental stages, this promotes more accurate and efficient in-depth exploration the functions of ncRNAs [8, 9]. For adipocyte differentiation, lots of ncRNAs have been shown their functional roles in many signaling pathways. Such as, mTOR [10], PI3K/Akt [11], MAPK [12], TGFβ [13] and Wnt [14] signaling pathways, and so on. CircRNAs as a type of ncRNA, characterized by a covalently closed-loop structure in which a downstream 5′ splice site (ss) is joined with an upstream 3′ ss, and often exhibit cell-type-specific and tissue-specific patterns [15, 16]. Natural circRNAs received high attention cause their efficient sponge roles [17]. For example, CircFUT10 promotes adipocyte proliferation and inhibits adipocyte differentiation in cattle via sponging let-7 [4]. Recently, identification lnc/circRNA-miRNA-mRNA competitive endogenous RNA network, find their interacts became a development direction in-depth research ncRNAs. For instance, study found that LncRNA CCDC26 interacts with CELF2 protein can enhance mice myeloid leukemia cell proliferation and invasion via the circRNA_ANKIB1/miR-195-5p/PRR11 Axis [18]. From the various studies on ncRNAs we can know that ncRNAs are involved in a broad regulatory network and play critical roles under different conditions [15, 19, 20]. While, there are very few related studies on the regulatory network of ncRNAs in the field of intramuscular adipocyte differentiation.

Here, we choose Jianzhou Goat, a goat breed in Chinese southwest, that well-received for its high meat percentage and high intramuscular fat content [16, 17]. Illumina HiSeq method was used to compare the transcriptomes data of goat intramuscular adipocytes differentiation. We purposed to find the differentially and highly expressed circRNAs between the early and late stages of intramuscular adipocytes differentiation. Explored their expression profiles and further predicted their possible interaction relationships. This work serves as a valuable genomic resource for in-depth exploration of the molecular mechanism of ncRNAs interaction network regulating adipocyte differentiation.

## Results

### CircRNAs mapped in goat intramuscular preadipocytes and adipocytes

**Fig. 1:**
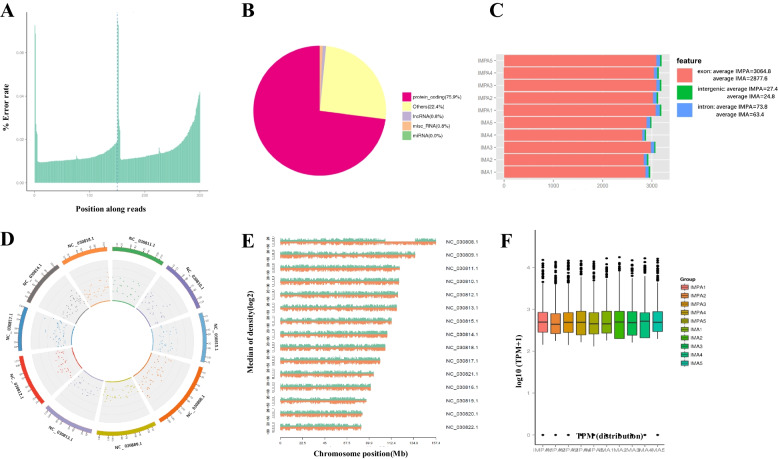
Identification of circRNAs in goat IMPA and IMA. **A** Sequencing error rate distribution, due to the consumption of chemical reagents, the error rate increases with the increase of the sequence length, which is one of the characteristics of illumina HiSeq, and the error rate per base is generally < 0.5%; **B** Clean reads distribution in known types of genes; **C** Source statistics of circRNAs in samples; **D** Density distribution of circRNAs on chromosomes, the outer edge is shown as the top 10 chromosomes, and the gray area in the middle is the distribution of 10,000 reads; **E** Distribution of the reads on each chromosome of the reference genome, sliding window is 1 kb and green is positive chain, red is negative chain; **F** Boxplots of the circRNAs expression levels in the sample, using TPM to normalize statistics

Using the illumina HiSeq high-throughput assay platform, we mapped 88–100 and 85–104 million clean reads in goat intramuscular preadipocytes (IMPA) and intramuscular adipocytes (IMA), respectively (Table 1), and the error rate along reads < 0.5% (Fig. 1 A). Comparison the circRNAs that mapped in clean reads with HISAT2 software. Among the known types of genes, we found that approximately 75.9% of the reads mapped to protein coding regions, 0.8% mapped to LncRNAs or miscellaneous RNA regions, and 22.4% of the reads were not mapped (Fig. 1 B). CircRNAs can be derived from exon or intron splicing, and we found exon-derived circRNAs were occupied the majority in this study (Fig. 1 C). In addition, the density of read circRNAs were counted in each chromosome, we showed the Top10 chromosomes in the genome (Fig. 1 D), and the total number of mappings on the chromosomes (Fig. 1 E). The longer the length of the entire chromosome, the more total mapped reads in the chromosome. We next normalized the circRNAs expression patterns of the samples using TPM density distribution map, and our results showed that the number of CircRNAs transcripts in IMPA were slightly less than IMA (Fig. 1 F).

**Table 1:** The RNA-seq mapping reads of goat reference genome

Samples	Raw Reads	Clean Reads	Error Rate	Mapped Reads	Mapping Ratio	Uniquely mapped	Uniquely Mapping Ratio
**IMA1**	**106,965,744**	**104,026,824**	**0.01%**	**58,826,296**	**56.55%**	**53,818,519 **	**51.74%**
**IMA2** **IMA3** **IMA4** **IMA5**	**97,739,340**	**95,783,582**	**0.01%**	**52,467,109**	**54.78%**	**49,088,892**	**51.25%**
**IMA3**	**100,355,022**	**98,296,238**	**0.02%**	**64,127,686**	**65.24%**	**59,783,468**	**60.82%**
**IMA4** **IMA3** **IMA4** **IMA5**	**87,081,698**	**85,127,438**	**0.02%**	**4,935,928**	**57.98%**	**46,064,921**	**54.11%**
**IMA5**	**93,900,560**	**92,052,240**	**0.01%**	**53,774,996**	**58.42%**	**49,952,000**	**54.26%**
**IMPA1**	**89,914,112**	**87,780,640**	**0.02%**	**80,670,979**	**91.9%**	**73,919,641**	**84.21%**
**IMPA2** **IMPA1**	**89,869,988**	**87,544,572**	**0.02%**	**70,588,932**	**80.63%**	**64,477,079**	**73.65%**
**IMPA3**	**86,513,066**	**84,676,776**	**0.02%**	**79,535,538**	**93.93%**	**72,354,034**	**85.45%**
**IMPA4** **IMPA1**	**93,072,522**	**91,173,148**	**0.01%**	**78,490,007**	**86.09%**	**71,794,446**	**78.75%**
**IMPA5**	**103,727,284**	**100,918,664**	**0.02%**	**96,226,399**	**95.3%**	**88,134,788**	**87.33%**

### Differentially expressed circRNAs of goat IMPA and IMA

**Fig. 2:**
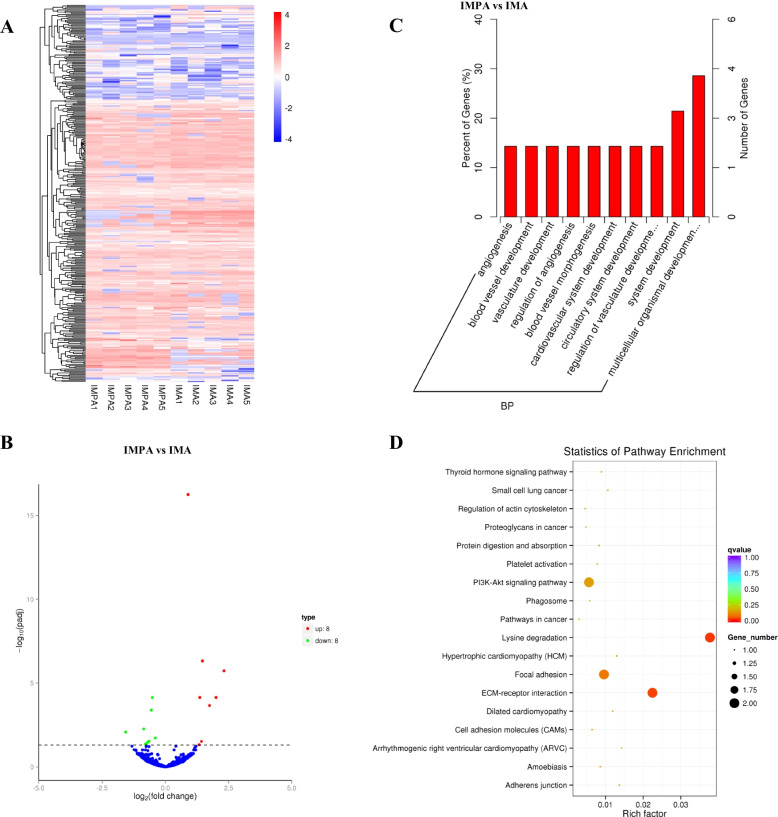
DE-circRNAs of goat IMPA and IMPA. **A** Hierarchical clustering diagram of DE-circRNAs, clustering as log10 (TPM + 1) value, red indicates high expression circRNAs, blue indicates low expression circRNAs; **B** Volcano plot of DE-circRNAs in IMPA and IMA, the screening condition is qvalue< 0.01; **C** GO enrichment histogram of the DE-circRNAs source gene; **D** KEGG enrichment scatter plot of the DE-circRNAs source gene

Based on log2 fold change and TPM density distribution of expression, the DE-circRNAs were identified using TMM normalization and DEGseq [18, 21]. For comprehensive observation, we union the differentially expressed CircRNA sets of each experimental sample, and cluster the CircRNAs with log10(TPM + 1) value from large to small according to the TPM value, the hierarchical clustering analysis showed that there were more high-expression CircRNAs in each samples (Fig. 2 A). Then, we compared the data of goat IMPA with IMPA, we found there were 8 downregulated and 8 upregulated circRNAs when selection filter condition was q-value< 0.01 (Fig. 2 B), and details of the circRNAs were shown in Table 2. To further analyze the potential functions of circRNAs, GO and KEGG were performed for the characterization of the identified circRNAs. For cellular components (Fig. S1), 2 GO terms were involved in collagen and extracellular matrix part. Regarding molecular function (Fig. S2), 2 GO terms were classified in protein binding and S-adenosylmethionine-dependent methyltransferase activity. For the biological process (Fig. S3), the dominant categories were multicellular organismal development, system development and regulation of angiogenesis and so on. Notably, the function annotation analysis showed that the DE-circRNAs were mainly enriched in multicellular organismal development biological process (BP) (Fig. 2 C). Furthermore, the KEGG pathway analysis of these DE-circRNAs showed that they were very likely to participate in Lysine degradation, ECM-receptor interaction, Focal adhesion and PI3K-Akt signaling pathway (Fig. 2 D). The GO and KEGG terms of host genes encoding DE-circRNAs (*P* < 0.05) were listed in Supplementary Material S1. Further analysis the enrichment results, we found that both circ_0005870 and circ_0000946 were existed in the Focal adhesion and PI3K-Akt signaling pathway, which are closely related to cell proliferation, differentiation and lipid metabolism.

**Table 2:** DE-circRNAs in goat IMPA and IMPA

Category	Gene symbol	Reference sequence	Spliced Length (nt)	Location on Chromosome	Chromosome Location	Log2 Fold change	***P-***value	Host Gene
**Up**	**circ_0008259**	**NC_030823.1**	**132**	**69,271,525 to 69,272,417**	**16**	**0.89937** **1**	**4.23E**^**− 20**^	**VASH2**
	**circ_0006499**	**NC_030819.1**	**667**	**35,520,125 to 35,552,054**	**12**	**1.4651** **RNA binding protein**	**7.32E**^**−10**^	**LMO7**
	**circ_0002854**	**NC_030812.1**	**324**	**64,143,968 to 64,145,981**	**5**	**2.3205** **isoform X1**	**4.29E**^**−09**^	**MYBPC1**
	**circ_0005870**	**NC_030818.1**	**432**	**105,098,460 to 105,103,883**	**11**	**2.0049**	**2.78E**^**−07**^	**COL5A1**
	**circ_0006511**	**NC_030819.1**	**309**	**35,548,438 to 35,552,054**	**12**	**1.3626**	**2.35E**^**−07**^	**LMO7**
	**circ_0011446**	**NC_030835.1**	**415**	**21,013,223 to 21,021,880**	**28**	**1.746**	**1.18E**^**−06**^	**MYPN**
	**circ_0010443**	**NC_030831.1**	**239**	**20,812,597 to 20,853,568**	**24**	**1.4274**	**0.00030055**	**FHOD3**
	**circ_0009924**	**NC_030829.1**	**255**	**10,094,532 to 10,113,699**	**22**	**1.3365**	**0.00059057**	**STAC**
**Down**	**circ_0005478**	**NC_030817.1**	**311**	**51,801,888 to 51,805,740**	**10**	**−0.51734**	**3.34E**^**−07**^	**SLTM**
	**circ_0009677**	**NC_030828.1**	**397**	**26,404,024 to 26,405,703**	**21**	**−0.55359** **enzyme 1**	**2.53E**^**− 06**^	**CEMIP**
	**circ_0005888**	**NC_030818.1**	**507**	**105,666,854 to 105,670,084**	**11**	**−0.8569** **factor 1 like**	**3.76E**^**− 05**^	**EHMT1**
	**circ_0000946**	**NC_030809.1**	**309**	**126,796,661 to 126,798,108**	**2**	**−1.5677** **protein**	**6.45E**^**−05**^	**ITGAV**
	**circ_0005860**	**NC_030818.1**	**425**	**102,883,876 to 102,885,038**	**11**	**−0.39615**	**0.00015792**	**CAMSAP1**
	**circ_0009231**	**NC_030826.1**	**305**	**61,732,411 to 61,736,148**	**19**	**−0.6421**	**0.00026862**	**CEP112**
	**circ_0008834**	**NC_030825.1**	**325**	**6,479,797 to 6,483,976**	**18**	**−0.70255**	**0.00036933**	**WWOX**
	**circ_0003129**	**NC_030813.1**	**207**	**115,901,669 to 115,924,848**	**6**	**−0.78362**	**0.00049942**	**WHSC1**

### Validation of DE-circRNAs using qRT-PCR combined with RNA-seq

**Fig. 3:**
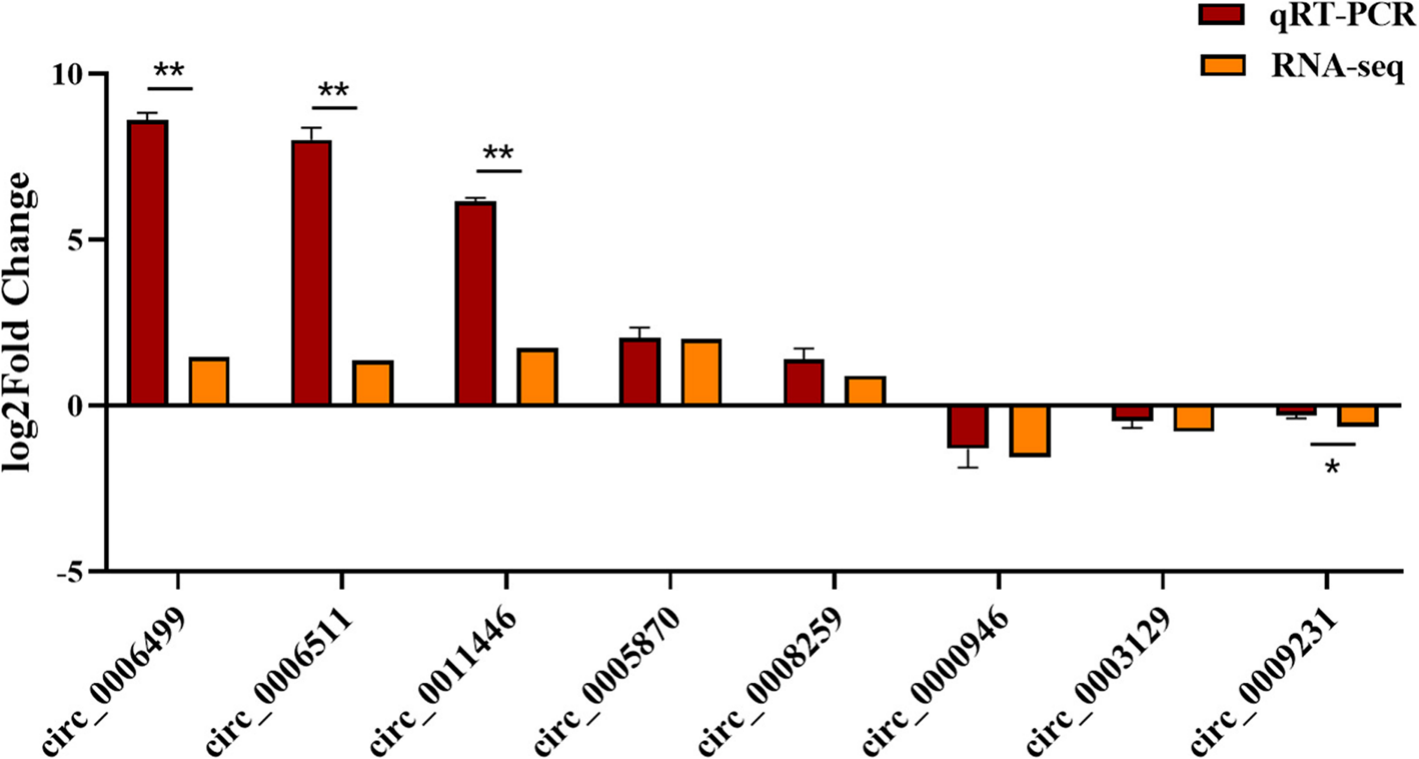
Validation of DE-circRNAs using qRT-PCR. The qRT-PCR data were analyzed using 2^−ΔΔCt^ method, and ubiquitously expressed transcript (UXT) was used as endogenous control. *P < 0.05, **P < 0.01

We selected 8 DE-circRNAs randomly to verify their expression levels by qRT-PCR method, the circPirmer were used to design amplification primers (Supplementary Material S2), the result shown in (Fig. 3). Our results of qRT-PCR were consistent with those of RNA-seq, circ_0009231, circ_0000946 and circ_0003129 were down regulated ncRNAs, and the circ_0006499, circ_0006511, circ_0011446, circ_0008259 and circ_0005870 were up regulated ncRNAs. Meanwhile, the expression level of circ_0006499, circ_0006511, circ_0011446 in qRT-PCR results were significantly higher than RNA-seq, the expression level of circ_0009231 in qRT-PCR result was significantly lower than RNA-seq.

### Identification of circRNA-miRNA network

**Fig. 4:**
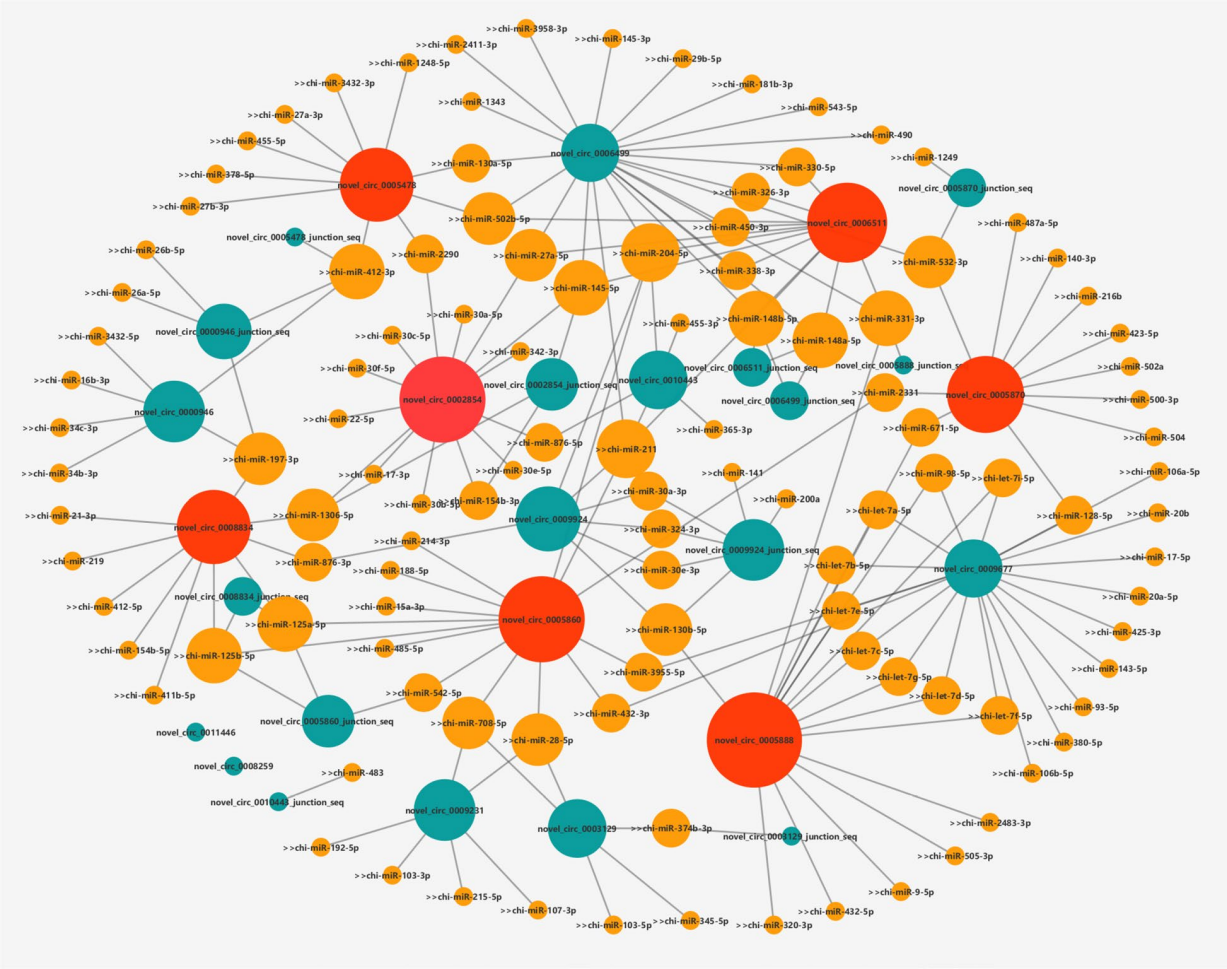
The interaction network of circRNA-miRNA. The red network nodes represent the circRNAs that existed in both Focal adhesion and PI3K-Akt signaling pathway, the green network nodes represent the DE-circRNAs in our research, orange network nodes represent the target miRNAs of the DE-circRNAs and larger nodes represent more interacting miRNAs

Remarkably, emerging studies have manifested that some circRNAs have miRNA binding sites, and function as miRNA sponges to regulate gene expression and play crucial roles in multiple cellular processes [22, 23]. To construct a circRNA-miRNA network, we used miRanda and TargetFinder softwares to predict miRNA binding sites for spliced circRNAs and the predicted results were listed in Supplementary Material S3. Based on the result we generated the circRNA-miRNA network diagram for the 8 DE-circRNAs, and the circ_0005870 and circ_0000946 that both existed in Focal adhesion and PI3K-Akt signaling pathways were marked with red. (Fig. 4).

### A circRNA-miRNA-mRNA network associated with goat intramuscular adipocyte differentiation

**Fig. 5:**
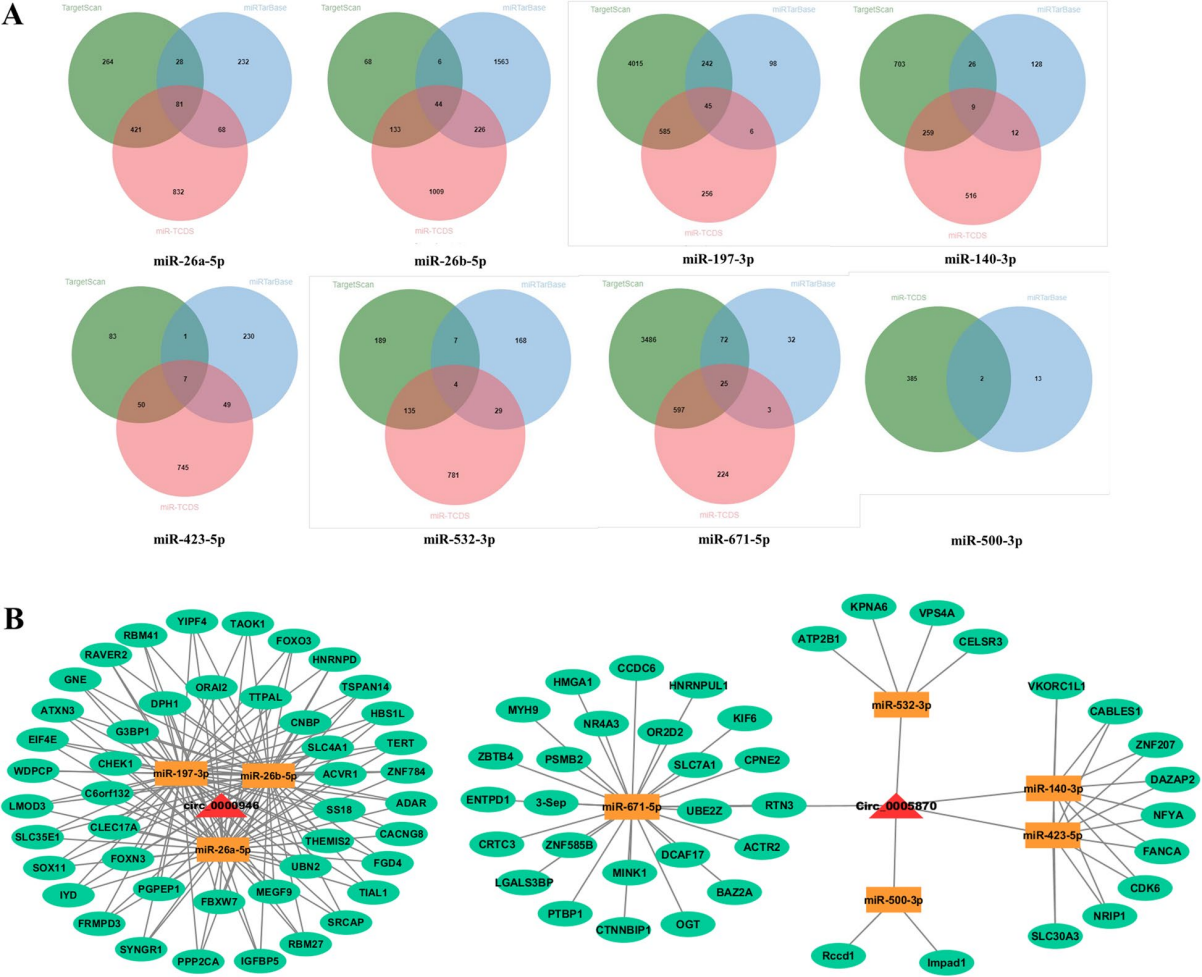
The circRNA-miRNA-mRNA interaction network. **A** Venn diagram showing the number of mRNAs in the databases that targeting the miRNAs; **B** The red triangle nodes represent the circRNAs that may having function roles in adipocyte differentiation, the orange square nodes represent the miRNAs that interacting with the circRNAs and the green network nodes represent the mRNAs we obtained from the databases

Two circRNAs, circ_0005870 and circ_0000946, were found existed in both focal adhesion [24, 25] and PI3K-Akt signaling pathways [11, 26] in this study, which given a great possibility of their functions in adipocyte differentiation. Therefore, we further performed prediction analysis to find the potential target genes of miRNAs that interacting with these two circRNAs. By overlapping the prediction results of three online databases, TargetScan, miRTarBase and miR-TCDS, we obtained the target mRNAs that may interact with the miRNAs (Fig. 5A, Supplementary Material S4), then, we generated the circRNA-miRNA-mRNA network diagram, based on the above results (Fig. 5B).

### GO and KEGG enrichment analysis of the target mRNA

**Fig. 6:**
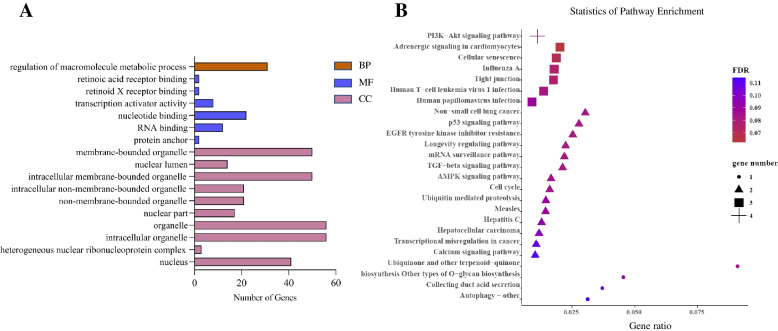
GO and KEGG enrichment analysis of the target mRNA. **A** GO enrichment histogram of the mRNAs; **B** KEGG enrichment scatter plot of the mRNAs, we showed top 25 here

To further explore the possible pathways that the circRNA-miRNA-mRNA interaction ways regulates goat intramuscular adipocyte differentiation in this study, we further performed GO and KEGG enrichment analysis on the target mRNAs (Fig. 6). The GO and KEGG analysis results were listed in Supplementary Material S5. From the enrichment analysis results, we found 31(46.2%) mRNAs were enriched in regulation of macromolecule metabolic process in BP (Fig. 6A), next, 5 of them were found enriched in the PI3K-Akt signaling pathway (FOXO3, PPP2CA, EIF4E, CDK6), AMPK signaling pathway (FOXO3, PPP2CA) and TGF-beta signaling pathway (ACVR1, PPP2CA), respectively (Fig. 6B). This result suggests the potential key roles of FOXO3, PPP2CA, EIF4E, CDK6 and ACVR1 in our circRNA-miRNA-mRNA interaction network.

## Discussion

Fat exists in almost all organisms. It is not only an important energy storage organ, but also has a regulatory effect on the physical support of tissues and organs, endocrine activity, energy balance and development [27, 28]. In animal husbandry field, the content of abdominal fat, subcutaneous fat and intramuscular fat of the industrialization animals are deemed as important indicator for the quality and flavor of meat [29–31]. Fat production can be divided into two stages, that are, mesenchymal stem cells turn into preadipocytes and terminal differentiation of the preadipocytes [32]. The procession of preadipocytes differentiation into mature adipocytes involves multiple cellular and signal transduction pathways, because, once preadipocytes have started the adipogenesis program, transcriptional cascades would inducing expression of metabolic genes and adipokines associated with the adipocyte phenotypes, such as Peroxisome proliferator-activated receptor γ (PPARγ), CCAAT-enhancer-binding protein α (C/EBPα), Krüppel-like factors (KLFs) and fatty-acid-binding protein-4 (Fabp4) and so on [33]. In addition, some extracellular signalings that acting upstream of transcription factors have been shown to regulate adipogenesis. For example, canonical Wnt-family proteins initiate signaling through β-catenin-dependent and -independent pathways, which inhibit adipocyte differentiation via blocking the expression of PPARγ and C/EBPα [34–36]. Signal transduction pathways closely related to adipogenesis are also Notch signalling, DLK1/PREF1 signalling, MAPK pathway, PI3K/AKT signalling and so on [37–40]. With the development of high-throughput RNA-seq technology, molecular mechanisms that regulate the adipocyte differentiation have been furthering described. And it is no doubt that exploring preadipocytes as a tool for precise regulation of adipocyte differentiation will promoting the development of precision medicine and the industrialization reform of edible meat quality in animal husbandry.

The specific functions of ncRNAs in regulating gene expression at the epigenetic, transcriptional and post-transcriptional levels suggested their important roles in a variety of biological processes [37, 41, 42]. In this study, illumina HiSeq high-throughput RNA-seq analysis was performed to identified and screened DE-circRNAs, 8 downregulated and 8 upregulated circRNAs were revealed in goat intramuscular adipocytes samples. From the KEGG enrichment analysis of the DE-circRNAs, we found that circ_0005870 and circ_0000946 were both enriched in the Focal adhesion and PI3K-Akt signaling pathways. Previous studies have demonstrated that focal adhesion kinase can induce mesenchymal stem cells (MSCs) differentiation [38]. Furthermore, same as multiple types of adipogenic induction, a study found that heparin promotes adipogenic differentiation by disrupting focal adhesions in immortalized and normal mouse marrow stromal cells by upregulating adipogenic genes [39, 40]. The PI3K-Akt intracellular signaling cascade is involving in many cellular regulation processes, and some evidences have indicated that PI3K/Akt pathway is a positive regulator of terminal adipocyte differentiation in mouse preadipocytes, moreover, phosphoinositide 3-kinase is also required for human adipocyte differentiation [26, 43, 44]. Based on these results, we hypothesized that circ_0005870 and circ_0000946 play an important role in the early differentiation of goat intramuscular preadipocytes.

Among ncRNAs, circRNAs are pronlly considered to be more stable due to the lack of 5′ to 3′ polarity and polyadenylation tail. Recent study found that, circ_0030042 regulates abnormal autophagy and protects atherosclerotic plaque stability by targeting eukaryotic initiation factor 4A-III, in high-fat-diet fed ApoE^−/−^ mice [45]. In adipose tissues from cachectic patients, circPTK2 competitively binding to miR-182-5p then promoted lipolysis and lipogenesis were inhibitied [46]. Based on circRNA-seq analysis, circBDP1 was proved to regulate bovine fat development by sponging miR-181b/miR-204 targeting Sirt1/TRARG [47]. In this study, we using miRanda and TargetFinder softwares predicting the miRNA binding sites for these DE-circRNA and a circRNA-miRNA interaction network for the DE-circRNAs was constructed by using Cytoscape software. From our interaction network, we found that circ_0000946 may have an interaction relationship with miR-412-3p, miR-197-3p, miR-26b-5p, miR-26a-5p, miR-3432-5p, miR-16b-3p, miR-34c -3p and miR-34b-3p. Among them, miR-197-3p [48], miR-26b-5p [49] and miR-26a-5p [50] have showed their correlation with adipocyte differentiation. For circ_0005870, we found it can have an interaction with miR-1249, miR-532-3p, miR-487a-5p, miR-140-3p, miR-216b, miR-423-5p, miR-502a, miR-500-3p, miR-504, miR-128-5p, miR-671-5p and miR-2331. Among them, miR-532-3p [51], miR-140-3p [50], miR-423-5p [52], miR-500-3p [53] and miR-671-5p [54] have showed their correlation with adipocyte differentiation.. In summary, our research indicated that the significantly DE circRNAs in goat preadipocytes might interact with some potential miRNAs.

In order to make our interaction network more hierarchically and characteristic, so, we selected circ_0005870 and circ_0000946, related to adipocyte differentiation, and their interaction miRNAs for further analysis. We first overlapped the prediction results from three online databases TargetScan, miRTarBase and miR-TCDS, obtained the potential target genes of the miRNAs, and generated the circRNA-miRNA-mRNA interaction network. Based on the predicted results, we next performed GO and KEGG enrichment analysis on these target mRNAs. The GO enrichment result showed that the mRNAs we found were all participated in macromolecule metabolic process. Three pathways, PI3K-Akt signaling pathway, AMPK signaling pathway and TGF-beta signaling pathway, got our attentions in KEGG enrichment result, cause they all having essential roles during adipocyte differentiation [26, 55, 56]. Then we obtained 5 key mRNAs in the circRNA-miRNA-mRNA network, FOXO3 [57], PPP2CA [58], EEIF4E [59], CDK6 [60] and ACVR1 [61], and they all showed valuable functions in fat-related research. The above results showed that the circRNA-miRNA-mRNA interaction network in this study can provide a reliable research idea for further exploring the mechanism of goat adipocyte differentiation. However, these findings are preliminary, and future research is needed to confirm them.

## Conclusions

Our finding revealed the expression profile and potential roles of circRNAs in goat intramuscular adipocytes. For adipocyte differentiation, we found circ_0005870 and circ_0000946 may play function roles in Focal adhesion and PI3K-Akt signaling pathway. By using online databases, we generated the final circRNA-miRNA-mRNA interaction network, and further showed 5 mRNAs, FOXO3, PPP2CA, EEIF4E, CDK6 and ACVR1, who existed in key signaling pathways in adipocyte differentiation. Taken together, our study explored the potential molecular mechanism of ncRNAs interaction network regulating adipocyte differentiation.

## Materials and methods

### The experiment flow in this study

Five samples of goat IMPA, induced 0d, and IMA, induced 3d, respectively, were performed Illumina HiSeq to identify the differentially expressed circRNAs, and the experimental strategy is shown in Supplementary material S6.

### Animals

The samples were collected from the longissimus dorsi muscle intramuscular fat of three healthy Jianzhou goat (male, 7 days old) that purchased from Jianyang Da Geda Animal Husbandry Co., Ltd. (Sichuan, China). Isolation procedures of goat intramuscular preadipocytes have been published previously [62, 63].

### Cell culture

The goat intramuscular preadipocytes were cultured in DMEM/F12 (Hyclone, USA) medium that containing 10% (v/v) fetal bovine serum (FBS, Hyclone, USA) and put in a humidified incubator with 5% CO2 at 37 °C for proliferation. To induce preadipocyte differentiation we replaced medium with adipogenic inducer of DMEM/F12 containing 10% FBS and 50 μmol•L-1 oleic acid when the preadipocytes proliferated to 90% of the cell culture dish [64]. For RNA extraction and sequencing, IMPA and IMA were collected on day 0 and day 3 after the proliferation medium was changed to induction medium, respectively.

### RNA-Seq library construction and sequencing analysis

Total RNA of IMPA and IMA was isolated using TRIzol reagent (TaKaRa, Otsu, Japan), and Nanodrop and agarose gel electrophoresis methods were used to detecte RNA purity and integrity. Afterwards, RNA concentrations were quantified using NanoDrop spectrophotometer (NanoDrop, Wilmington, USA) and Agilent 2100 Bioanalyzer (Agilent, Santa Clara, USA). Before constructing the RNA-seq libraries, samples were treated with epicentre Ribo-Zero™ Kit (Epicentre, Madison, USA) to remove Ribosomal RNA. Library preparation and Illumina HiSeq analysis process were described in previous study [65]. The raw reads were analysised using High Performance Computing (HPC) and the quality assessment data was listed in Supplementary material S7.

### NcRNAS identification and the expression levels analysis

The raw reads obtained by sequencing were filtered with removed the low-quality reads to obtain clean reads. To identify reliable circRNAs, we used two software packages “find_circ” and “CIRI2” to identify and detected the circRNAs, separately [66, 67]. Combining the circRNAs identified by find_circ and CIRI2, the candidate circRNAs in “find_circ” group with read count ≥2were selected, and then intersection of the two identified results based on the location of the circRNAs in the chromosome. The expression statistics of known and novel circRNAs in each sample was performed, and the raw counts were normalized using TPM [68]. Normalized expression level = (readCount*1,000,000) / libsize (libsize is the sum of the circRNA read count). For small RNA libraries, we used novoaligen software to align clean reads with the miRBase database to identify known miRNAs, and used mirDeep software to predict Novel miRNAs, and then used novoaligen and samtools software for quantitative analysis, using RPM (reads per million) for standardization [69–72].

### RNA extraction and qRT-PCR

Total RNA was extracted using TRIzol (TaKaRa, Otsu, Japan), and stored at − 80 °C. The mRNA was reverse transcripted using a Revert Aid First Strand cDNA Synthesis Kit (TaKaRa, Otsu, Japan) according to manufacturer instructions. The reaction volume for qRT-PCR was 20 μL that consisted of 1 μL cDNA, 1 μL reverse and forward primers, 7 μL double-distilled water, and 10 μL TB Green™ Premix Ex Taq™ II (TaKaRa, Otsu, Japan). The relative expression levels were determined by the 2^−ΔΔCt^ method.

### Gene ontology and pathway analysis

We used GO analysis (http://www.geneontology.org/) to characterize circRNA-hosting and miRNA-hosting genes. GO terms provide information about the biological processes, cellular components and metabolic pathways that genes involved in. Meanwhile, the KEGG (http://www.kegg.jp/) pathway analysis was also performed to obtain insight into the molecular interactions of the differential expression circRNAs and miRNAs [73–75]. Targetscan (https://www.targetscan.org/vert_80/), miRTarBase (https://maayanlab.cloud/Harmonizome/dataset/MiRTarBase+microRNA+Targets) and miRT-CDS (http://www.microrna.gr/microT-CDS) were used to predict the miRNAs target genes.

### Statistical analysis of data

The RNA-seq data were all performed Benjamini & Hochberg False Discovery Rate (FDR) correction. qRT-PCR data were expressed with log2 fold change value and visualized using GraphPad Prism 8 software. The circRNA-miRNA and circRNA-miRNA-mRNA interaction network were visualized using Cytoscape software. Visualization of volcano plots and bubble plots using ggplot2 and ImageGP (http://www.ehbio.com/ImageGP/index.php/Home/Index/index.html).

## Supplementary Information

**Additional file 1: Fig. S1.** GO enrichment histogram of the DE-circRNAs source genes for cellular components. **Fig. S2.** GO enrichment histogram of the DE-circRNAs source genes for molecular function. **Fig. S3.** GO enrichment histogram of the DE-circRNAs source genes for biological process.

**Additional file 2: Supplementary Material S1.** The GO terms of host genes encoding DE-circRNAs.

**Additional file 3: Supplementary Material S2.** The expression of circRNAs in each sample was normalized by TPM.

**Additional file 4: Supplementary Material S3.** The predicted results of miRNA-circRNAs binding pair.

**Additional file 5: Supplementary Material S4.** The miR-140-3p target genes prediction results in TargetScan. Common elements in TargetScan miRTarBase miR-TCDS. The miR-671-5p target genes prediction results in TargetScan. The miR-532-3p target genes prediction results in TargetScan. The miR-500-3p target genes prediction results in TargetScan. The miR-423-5p target genes prediction results in TargetScan. The miR-26b-5p target genes prediction results in TargetScan. Common elements in TargetScan miRTarBase miR-TCDS.

**Additional file 6: Supplementary Material S5.** Top 20 of the KEGG Pathway enrichment analysis.

**Additional file 7: Supplementary material S6.** Schematic diagram of the experimental flow. The first horizontal arrow indicates the process of preadipocytes differentiation into adipocytes. The vertical arrows indicate the experimental flow. Diagram indicate the sample model establishment, experiment principles, data acquisition and data analysis.

**Additional file 8: Supplementary Material S7.** The data quality assessment table.

## Abbreviations

IMPA: intramuscular preadipocytes
IMA: intramuscular adipocytes
ncRNAs: noncoding RNAs
miRNAs: microRNAs
circRNAs: circular RNAs
DE-circRNAs: differentially expressed circRNAs
KEGG: Kyoto Encyclopedia of Genes and Genomes
GO: Gene Ontology
qRT-PCR: real-time fluorescent quantitative PCR.
