# Supplementary material for: Key circRNAs from goat: discovery, integrated regulatory network and their putative roles in the differentiation of intramuscular adipocytes
Source: BMC Genomics. 2023 Jan 28;24:51. doi: 10.1186/s12864-023-09141-7 (PMC9883971; doi:10.1186/s12864-023-09141-7)
Supplement: Supplementary file 1 — Additional file 1: Fig. S1. GO enrichment histogram of the DE-circRNAs source genes for cellular components. Fig. S2. GO enrichment histogram of the DE-circRNAs source genes for molecular function. Fig. S3. GO enrichment histogram of the DE-circRNAs source genes for biological process. [file 12864_2023_9141_MOESM1_ESM.docx]

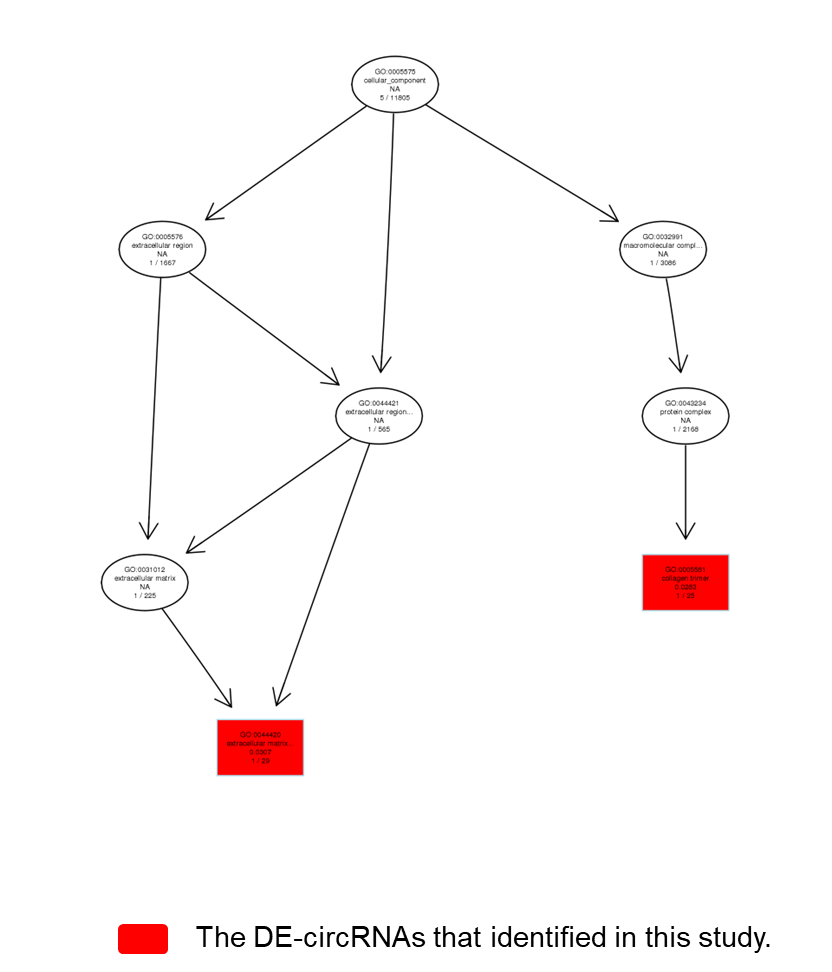


Figure S1: GO enrichment histogram of the DE-circRNAs source genes for cellular components.


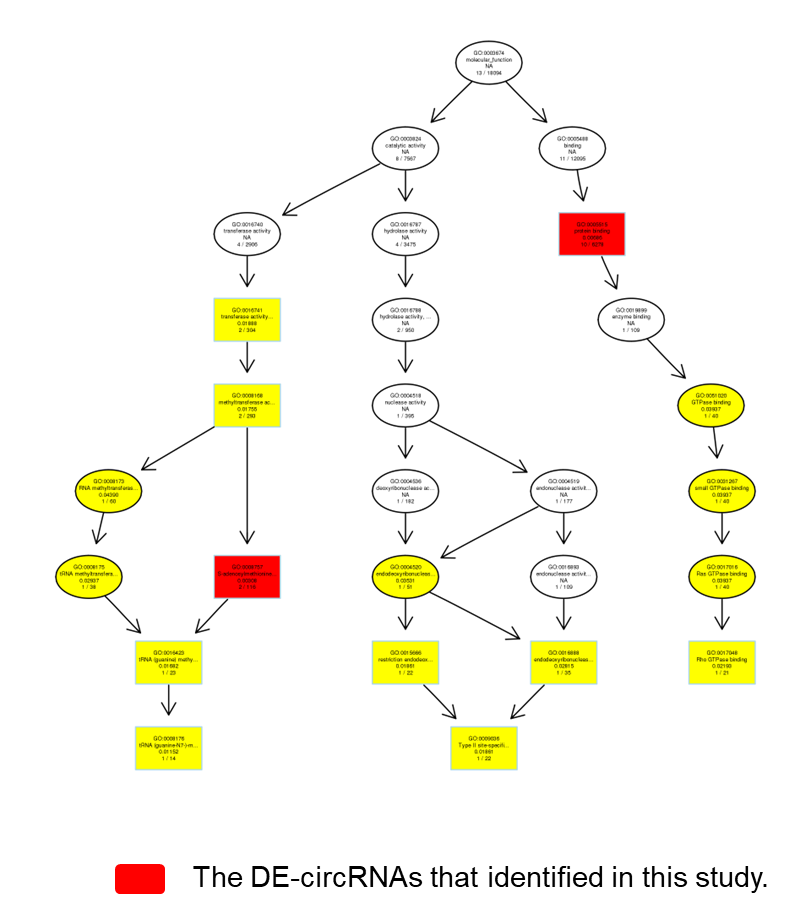


Figure S2: GO enrichment histogram of the DE-circRNAs source genes for molecular function.


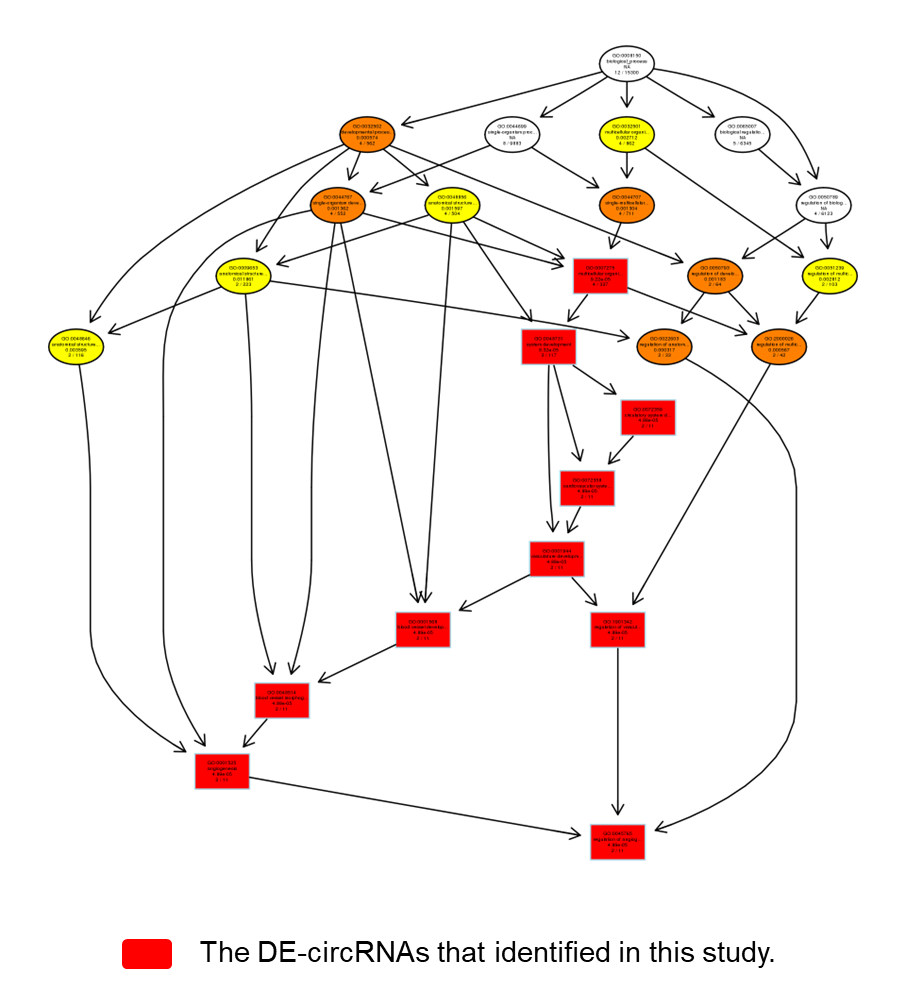


Figure S3: GO enrichment histogram of the DE-circRNAs source genes for biological process.
