# Supplementary material for: Key circRNAs from goat: discovery, integrated regulatory network and their putative roles in the differentiation of intramuscular adipocytes
Source: BMC Genomics. 2023 Jan 28;24:51. doi: 10.1186/s12864-023-09141-7 (PMC9883971; doi:10.1186/s12864-023-09141-7)
Supplement: Supplementary file 7 — Additional file 7: Supplementary material S6. Schematic diagram of the experimental flow. The first horizontal arrow indicates the process of preadipocytes differentiation into adipocytes. The vertical arrows indicate the experimental flow. Diagram indicate the sample model establishment, experiment principles, data acquisition and data analysis. [file 12864_2023_9141_MOESM7_ESM.docx]

**
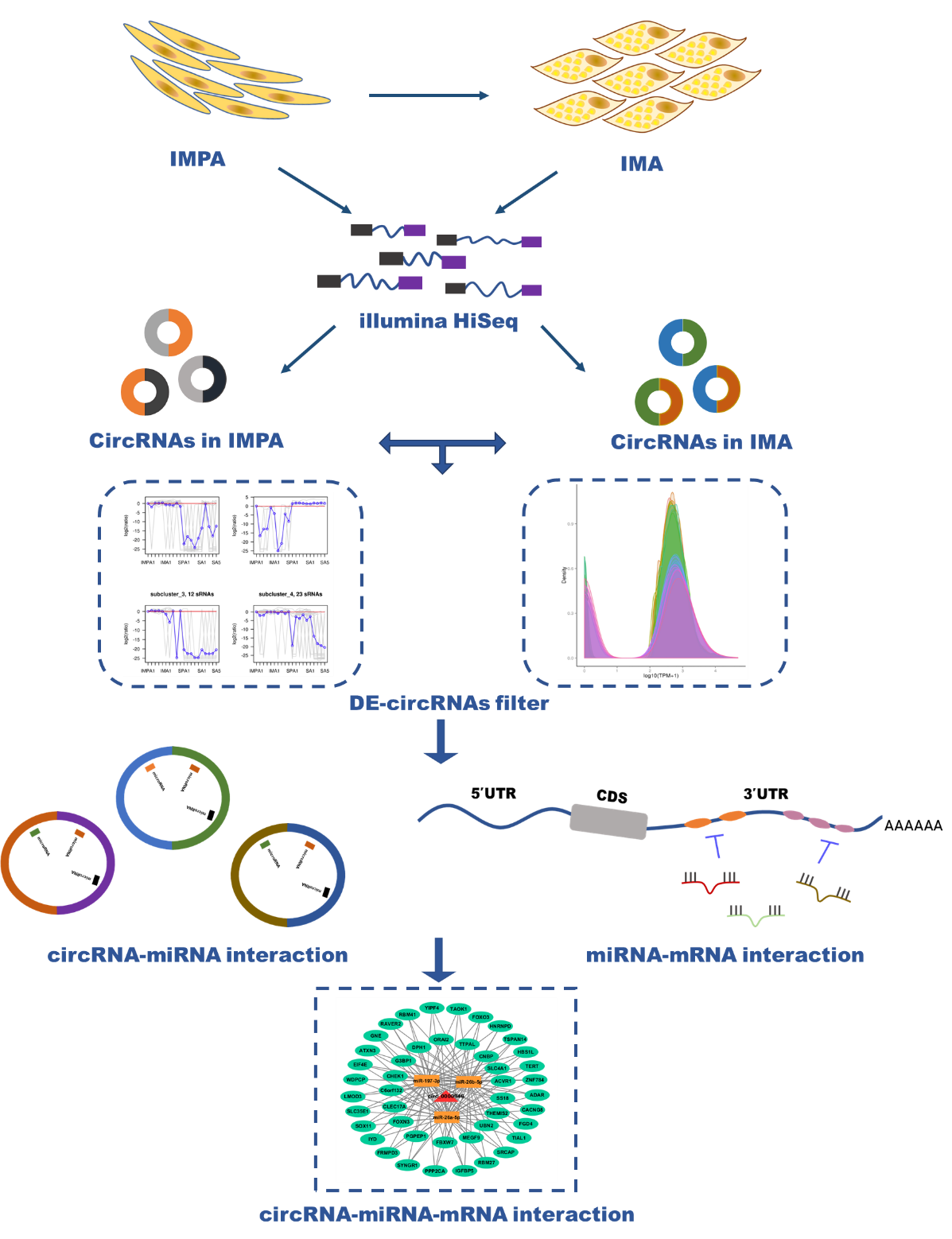
**

**Supplementary material S6: Schematic diagram of the experimental flow.** The first horizontal arrow indicates the process of preadipocytes differentiation into adipocytes. The vertical arrows indicate the experimental flow. Diagram indicate the sample model establishment, experiment principles, data acquisition and data analysis.
